# Schizophrenia-associated alterations in fecal mycobiota and systemic immune dysfunction: a cohort study of elderly Chinese patients

**DOI:** 10.3389/fimmu.2025.1607739

**Published:** 2025-07-21

**Authors:** Zongxin Ling, Yiwen Cheng, Xia Liu, Xiaocui Xu, Lingbin Wu, Li Shao, Zhangcheng Zhu, Wenwen Ding, Qinghai Song, Longyou Zhao, Guolin Jin

**Affiliations:** ^1^ Collaborative Innovation Center for Diagnosis and Treatment of Infectious Diseases, State Key Laboratory for Diagnosis and Treatment of Infectious Diseases, National Clinical Research Center for Infectious Diseases, the First Affiliated Hospital, School of Medicine, Zhejiang University, Hangzhou, Zhejiang, China; ^2^ Department of Intensive Care Unit, the First Affiliated Hospital, School of Medicine, Zhejiang University, Hangzhou, Zhejiang, China; ^3^ Department of Anesthesiology, Affiliated Hospital of Nantong University, Nantong, Jiangsu, China; ^4^ Department of Psychiatry, Lishui Second People’s Hospital, Lishui, Zhejiang, China; ^5^ School of Clinical Medicine, The Affiliated Hospital of Hangzhou Normal University, Hangzhou, Zhejiang, China; ^6^ Department of Preventive Medicine, School of Public Health and Management, Wenzhou Medical University, Wenzhou, Zhejiang, China

**Keywords:** Candida, gut mycobiota, immune dysfunction, Purpureocillium, schizophrenia

## Abstract

Schizophrenia (SZ) is a severe psychiatric disorder with a complex etiology involving both genetic and environmental factors. Emerging evidence highlights the role of gut microbiome dysbiosis in SZ, yet the fungal component (mycobiota) remains largely unexplored. This study aimed to evaluate the gut mycobiota using internal transcribed spacer 1 (ITS1) amplicon sequencing and assess host immune responses via multiplex immunoassays in 87 elderly SZ patients and 64 age- and gender-matched healthy controls (HCs). We observed significant increases in fungal α-diversity and richness, along with altered β-diversity in SZ patients. Specifically, there was an elevated Basidiomycota/Ascomycota ratio, with enrichment of *Candida*, *Aspergillus*, and *Saccharomyces*, coupled with a depletion of *Purpureocillium*. Enterotype analysis revealed a shift from *Purpureocillium*-dominant (E1) to *Candida*-dominant (E2) communities in SZ. Notably, key fungal species, such as *S. cerevisiae* and *P. lilacinum*, were correlated with systemic immune dysfunction. Our receiver operating characteristic (ROC) analysis indicated that these fungal species could effectively distinguish SZ patients from HCs, suggesting their potential as non-invasive biomarkers for SZ diagnosis. In conclusion, this study demonstrates significant alterations in the gut mycobiota and immune dysfunction in elderly SZ patients, suggesting that mycobiota dysbiosis may contribute to SZ pathogenesis through immune modulation, offering new avenues for potential biomarkers and therapeutic interventions.

## Introduction

Schizophrenia (SZ), a complex and debilitating psychiatric disorder, is characterized by a range of symptoms, including cognitive dysfunction, hallucinations, delusions, and social withdrawal (1). Individuals with SZ face a 2- to 2.5-fold increased risk of premature mortality compared to the general population, with a life expectancy reduction of 15 to 20 years (2), making it a significant public health concern. While its etiology remains unclear, accumulating evidence suggests that both genetic and environmental factors contribute to the onset and progression of the disease (3, 4). One promising area of investigation in SZ research is the role of the gut microbiome, specifically the microbial communities within the gastrointestinal tract, and their potential impact on brain function and immune regulation.

The gut microbiota comprises a diverse range of microorganisms, including bacteria, viruses, archaea, and fungi, all of which are essential for maintaining overall health. Fungi, often overlooked, are a critical part of this microbial ecosystem. Recent studies have emphasized the importance of the gut mycobiota (the fungal component of the microbiota) in health and its potential involvement in various diseases (5, 6). Gut fungi not only influence gut functions but also impact the physiological processes of other vital extraintestinal organs, including brain (5, 7). Dysbiosis of the gut mycobiota, which refers to an imbalance in the diversity and composition of the gut fungal community, has been associated with several neuropsychiatric disorders such as SZ (8). However, the alterations of gut mycobiota profiles in SZ remains largely unexplored.

The gut microbiota communicates with the central nervous system via the gut-brain axis, a complex pathway of bidirectional signaling between the gut and the brain. This communication modulates not only local immune responses within the gut but also systemic immune responses that can affect brain function and behavior. Research has linked gut bacteriota dysbiosis to a variety of neuropsychiatric disorders, including SZ (9–13). In our previous studies, we observed immune dysfunction in individuals with SZ, characterized by altered cytokine profiles and systemic inflammation, suggesting chronic low-grade inflammation (9, 10). Correlation analyses have indicated that changes in the gut microbiota can drive immune dysregulation in SZ, potentially contributing to neuroinflammation and worsening cognitive and psychiatric symptoms. Evaluation of the gut mycobiota has revealed that fungal communities are not only altered in disease but also play a role in maintaining gut homeostasis and influencing systemic immunity (8, 13, 14). While research on the gut mycobiota in health and disease is increasing, studies on its impact in SZ remain limited.

In the current study, we recruited 151 elderly participants, comprising 87 individuals diagnosed with SZ and 64 age- and sex-matched healthy controls (HCs), to explore the gut mycobiota profiles through fungal-specific internal transcribed spacer 1 (ITS1) amplicon sequencing. Additionally, we assessed serum immune markers utilizing the multiplex Bio-Plex technology. By investigating fecal fungal dysbiosis and its potential association with systemic immune dysfunction, we hope to enhance our understanding of the role of gut fungi in the pathophysiology of SZ and their interaction with immune system alterations. Identification of specific biomarkers and therapeutic targets associated with the gut mycobiota could offer novel insights into potential strategies for modulating the gut mycobiota to alleviate or treat SZ.

## Materials and methods

### Participants’ enrollment and sample collection

This study utilized the same cohort as in our previous research (9). Specifically, we recruited elderly Chinese SZ patients (aged ≥62 years) and cognitively HCs, matched by age and gender, from Lishui, Zhejiang Province, China, between June and November 2020. The study protocol was reviewed and approved by the Ethics Committee of the Lishui Second People’s Hospital (approval reference no.: 20180705-1). Prior to enrollment, written informed consent was obtained from all participants or their legally authorized caregivers. The inclusion and exclusion criteria, as well as the demographic and clinical characteristics of the participants, were aligned with the specifications detailed in our prior research (9).

Approximately 2g of fresh fecal samples were collected using standardized sterile collection tubes and stored at −80°C within 15 minutes of collection to preserve microbial integrity. Fasting blood samples were collected from participants in the early morning, processed within 15 minutes, and stored at −80°C until further use.

### ITS sequencing

Fungal genomic DNA was extracted from 300 mg of homogenized feces using a QIAamp^®^ DNA Stool Mini Kit (QIAGEN, Hilden, Germany) according to the manufacturer’s instructions, with additional glass-bead beating performed on a Mini-beadbeater (FastPrep; Thermo Electron Corporation, Boston, MA, USA). Fungal amplicon libraries targeting the ITS1 region (ITS1F: 5’- CTTGGTCATTTAGAGGAAGTAA-3’; ITS2R: 5’- GCTGCGTTCTTCATCGATGC-3’) were constructed by technical staff at Hangzhou KaiTai Bio-lab, followed by sequencing on the Illumina NovaSeq 6000 PE250 platform (15).

### Bioinformatic analysis

Sequence analyses were performed using QIIME2 (v2020.11) and its plugins with default settings. Non-biological sequences (adapter and barcode) were removed and trimmed using the Cutadapt plugin. Amplicon sequence variants (ASVs) were generated by denoising and performing quality control, including chimera removal, through the DADA2 plugin (16). Taxonomy was assigned to ASVs using the UNITE dynamic database (Release 9.0, http://unite.ut.ee/index.php). Sample reads were normalized to ensure consistent sequencing depth. Fungal α- and β-diversity, community composition, and correlation analyses among key functional fungi were conducted as previously described for bacterial microbiota (9, 17, 18). Gut fungal enterotypes were employed to identify distinct fungal communities across the samples. Additionally, enzyme classification (EC) numbers and MetaCyc pathway predictions were generated based on the ASVs using PiCRUSt2 (19, 20). Fungal function was further predicted based on the OTU table using the FUNGuild algorithm (21). To assess the discriminative power of key functional fungal taxa, Random Forest classification was applied, with Mean Decrease Gini used to evaluate variable importance. Receiver operating characteristic (ROC) analysis was performed to evaluate the ability of key functional fungi to discriminate SZ from HCs using the OECloud tools at https://cloud.oebiotech.com.

### Multiplex cytokine analysis

Cytokine analysis was performed to assess the systemic immune function of participants using the Bio-Plex Pro™ Human Cytokine 27-plex assay kit (Bio-Rad, CA, USA; catalog no. M500KCAF0Y) (9, 17, 18, 22, 23). The assay measured 16 cytokines, 6 chemokines, and 5 growth factors using the Bio-Plex^®^ 200 System (Bio-Rad Laboratories, Inc.), following the manufacturer’s guidelines. Serum samples were diluted fourfold with sample diluent buffer before analysis. Cytokine concentrations were determined based on standard curves and expressed in pg/mL using Bio-Plex Manager v5.0 software. The assay showed reproducibility with CVs of 5-8%. Quality control measures included validation of standard curves, dynamic range checks, and controls to ensure assay specificity. Outlier values were flagged, and cytokine concentrations below the limit of detection (LOD) were assigned half the LOD value to maintain data integrity. These steps ensured the reliability and accuracy of the results.

### Statistical analysis

Differences were assessed using various statistical tests, including White’s nonparametric *t*-test, independent *t*-test, or Mann-Whitney *U*-test, depending on data distribution and assumptions. Correlations were analyzed using Spearman’s rank correlation test. Statistical analyses were conducted using SPSS v24.0 (SPSS Inc., Chicago, IL) and Statistical Analysis of Metagenomic Profiles (STAMP) v2.1.3. Graphical representations were generated with R packages and GraphPad Prism v6.0. Predictive power was assessed through ROC curve analysis and area under the curve (AUC). ROC curve analysis is a robust method for performance evaluation, with AUC values providing insight into discriminatory power. All significance tests were two-sided, and p-values were adjusted for multiple testing using the Benjamini-Hochberg method to control the False Discovery Rate (FDR). A threshold of FDR < 0.05 was considered statistically significant.

### Accession number

The sequence data from this study are deposited in the GenBank Sequence Read Archive (PRJNA1243042).

## Results

### Altered overall structure of the fecal mycobiota in Chinese patients with SZ

**Figure 1:**
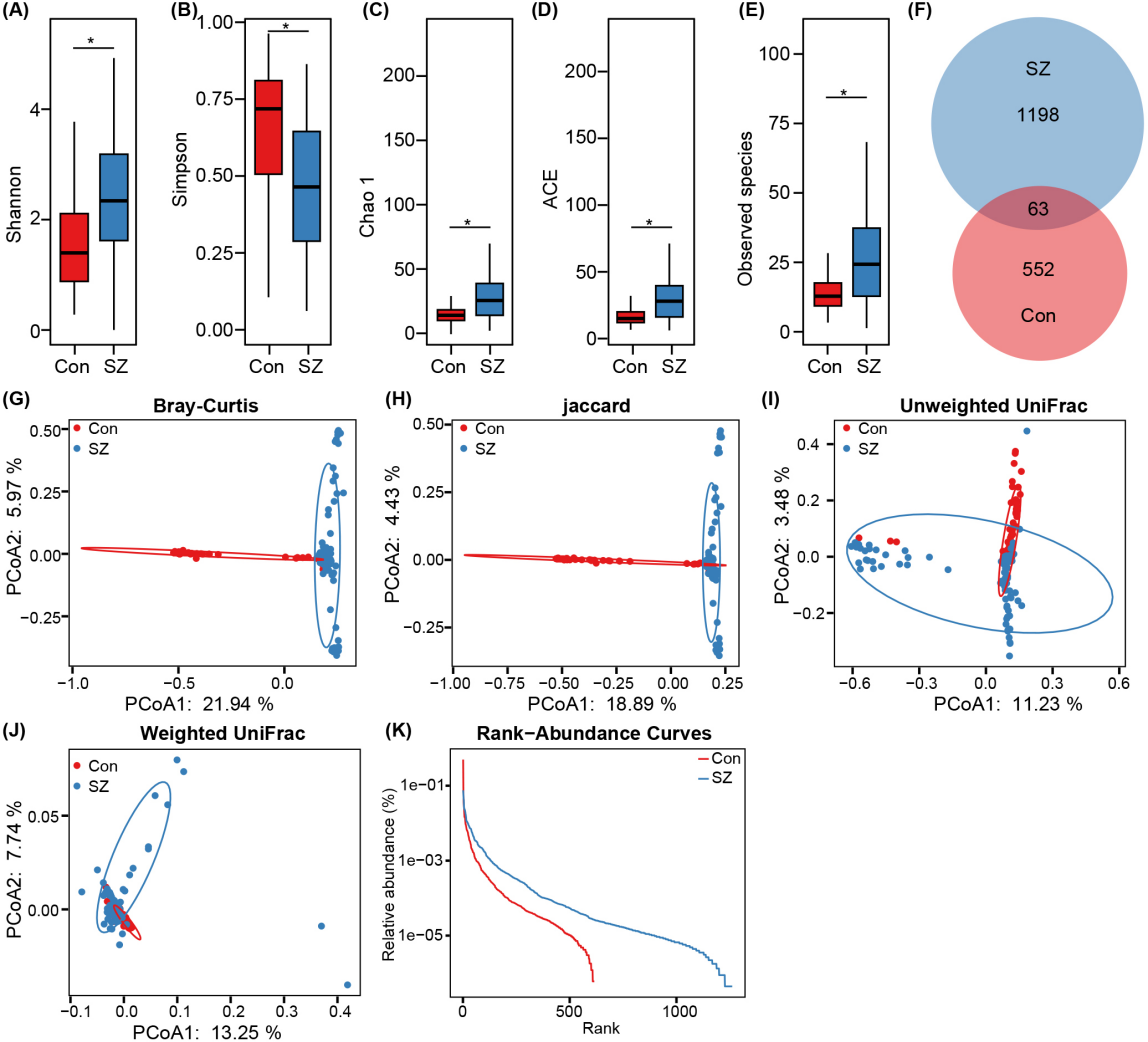
Comparison of the fecal fungal diversity and richness between SZ patients and healthy controls. **(A–E)** α-diversity indices (Shannon, and Simpson) and richness indices (Chao1, ACE, and observed species) were used to assess the overall structure of the fecal mycobiota, with data presented as mean ± standard deviation. Unpaired two-tailed t-tests were used for inter-group comparisons. **(F)** Venn diagram showing the overlap of amplicon sequence variants (ASVs) in the fecal mycobiota of SZ patients and healthy controls. **(G–J)** Principal coordinate analysis (PCoA) plots illustrating β-diversity of individual fecal mycobiota based on Bray–Curtis, unweighted UniFrac, and weighted UniFrac distances, with each symbol representing a sample. **(K)** The rank-abundance curve of fungal ASVs from both groups indicates a higher presence of low-abundance ASVs in the fecal mycobiota of SZ patients compared to healthy controls. *p < 0.05.

After successfully constructing fungal amplicon libraries, a final sample size of 87 SZ patients and 64 HCs was established for comprehensive fecal mycobiota analysis. After ITS1 sequencing, we obtained 9,613,219 high-quality sequence reads (6,824,365 for SZ and 2,788,854 for HCs) from 11,326,564 raw sequence reads, with an average of 63,663 reads per sample. To ensure consistent sequencing depth across all samples, we normalized each sample to 26,070 reads for the subsequent analysis. Across the entire cohort, we identified a total of 1,813 ASVs with 615 ASVs in the HC group and 1,261 ASVs in the SZ group. Fecal mycobiota diversity was compared between SZ patients and HCs based on the relative ASVs table (**Figure 1**). The fungal α-diversity indices, including Shannon and Simpson, were significantly higher in SZ patients, while the richness indices such as Chao1, ACE, and observed species were also significantly increased in SZ patients compared to HCs (p < 0.05; **Figures 1A–E**). The Venn diagram further revealed a higher number of unique fungal phylotypes in SZ patients compared to HCs (**Figure 1F**). For β-diversity analysis, principal coordinates analysis (PCoA) was performed using Bray-Curtis, jaccard, unweighted UniFrac, and weighted UniFrac algorithms. Significant differences in fecal fungal β-diversity were observed between SZ patients and HCs (ADONIS test: p = 0.001; **Figures 1G–J**), despite considerable interindividual variations within both groups. Additionally, the rank abundance curve demonstrated the species richness and evenness of microbial communities across the groups, indicating that the SZ group had higher species richness and evenness compared to the control group (**Figure 1J**). Collectively, these results suggest an altered fecal mycobiota structure in individuals with SZ.

### Fecal mycobiota dysbiosis in patients with SZ

**Figure 2:**
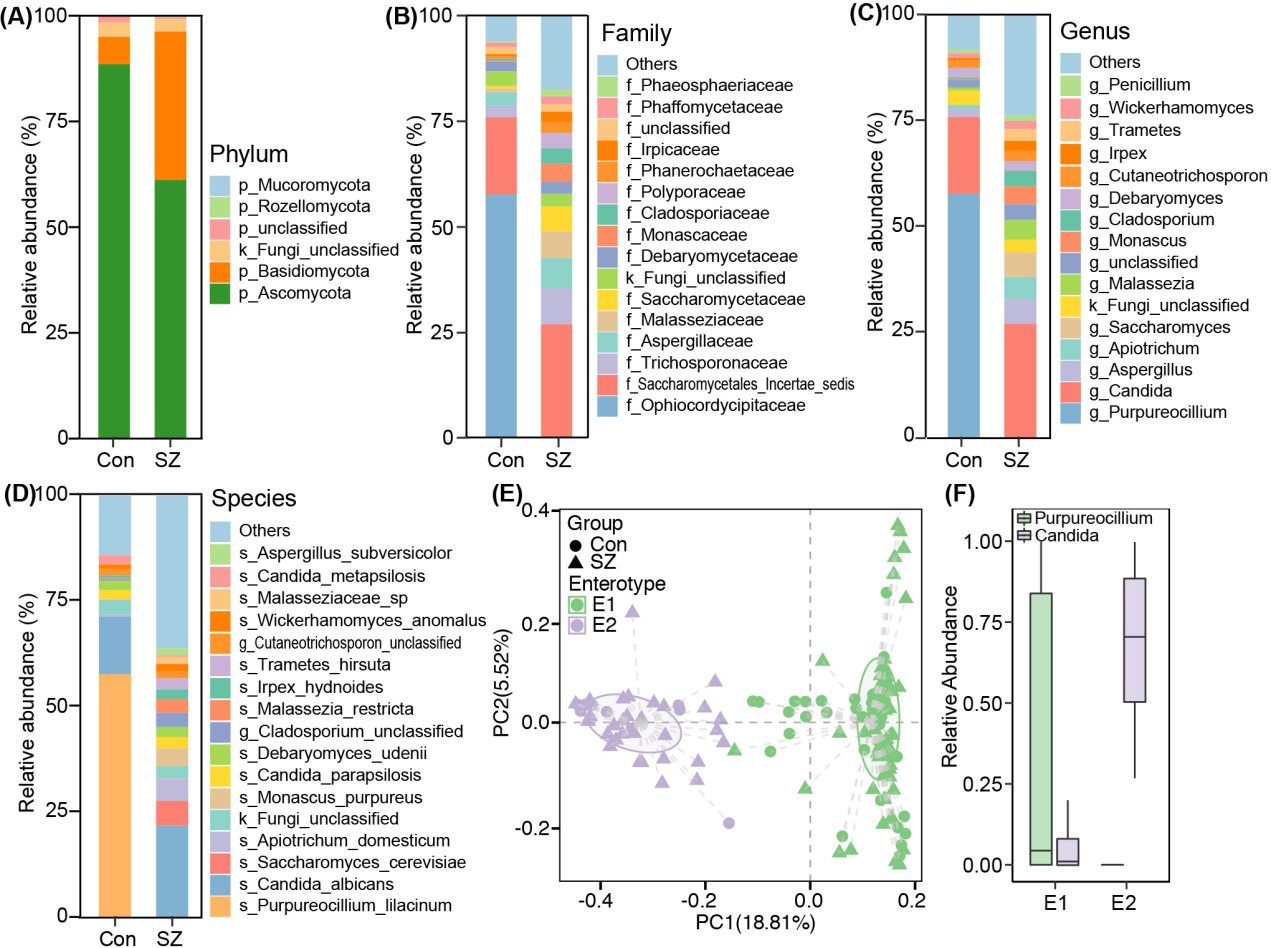
Fecal mycobiota compositions of in SZ patients and healthy controls. **(A)** Phylum; **(B)** Family; **(C)** Genus; **(D)** Species; **(E)** PCoA plot showing two enterotypes; **(F)** Relative abundances of representative genera of enterotypes.

After taxonomic classification of the fecal mycobiota, we identified 4 phyla, 123 families, 204 genera, and 301 species from both cohorts. **Figure 2** depicts the fecal mycobiota landscape at the phylum (**Figure 2A**), family (**Figure B**), genus (**Figure C**), and species (**Figure 2D**) levels. The dominant phyla were Ascomycota and Basidiomycota, together accounting for over 96% of the total sequences analyzed. Enterotypes were categorized within the extensive study population using previously established methods (24). The fecal mycobiota could be categorized into two enterotypes, E1 and E2 at the genus level (**Figure 2E**). The E1 enterotype was dominated by *Purpureocillium*, while the E2 enterotype was dominated by *Candida* (**Figure 2F**). In the control group, 84.4% of samples (54/64) were classified as E1 enterotype, while 65.5% of samples (57/87) in the SZ group belonged to the E1 enterotype, indicating a significant difference in microbiota composition between the two groups.

**Figure 3:**
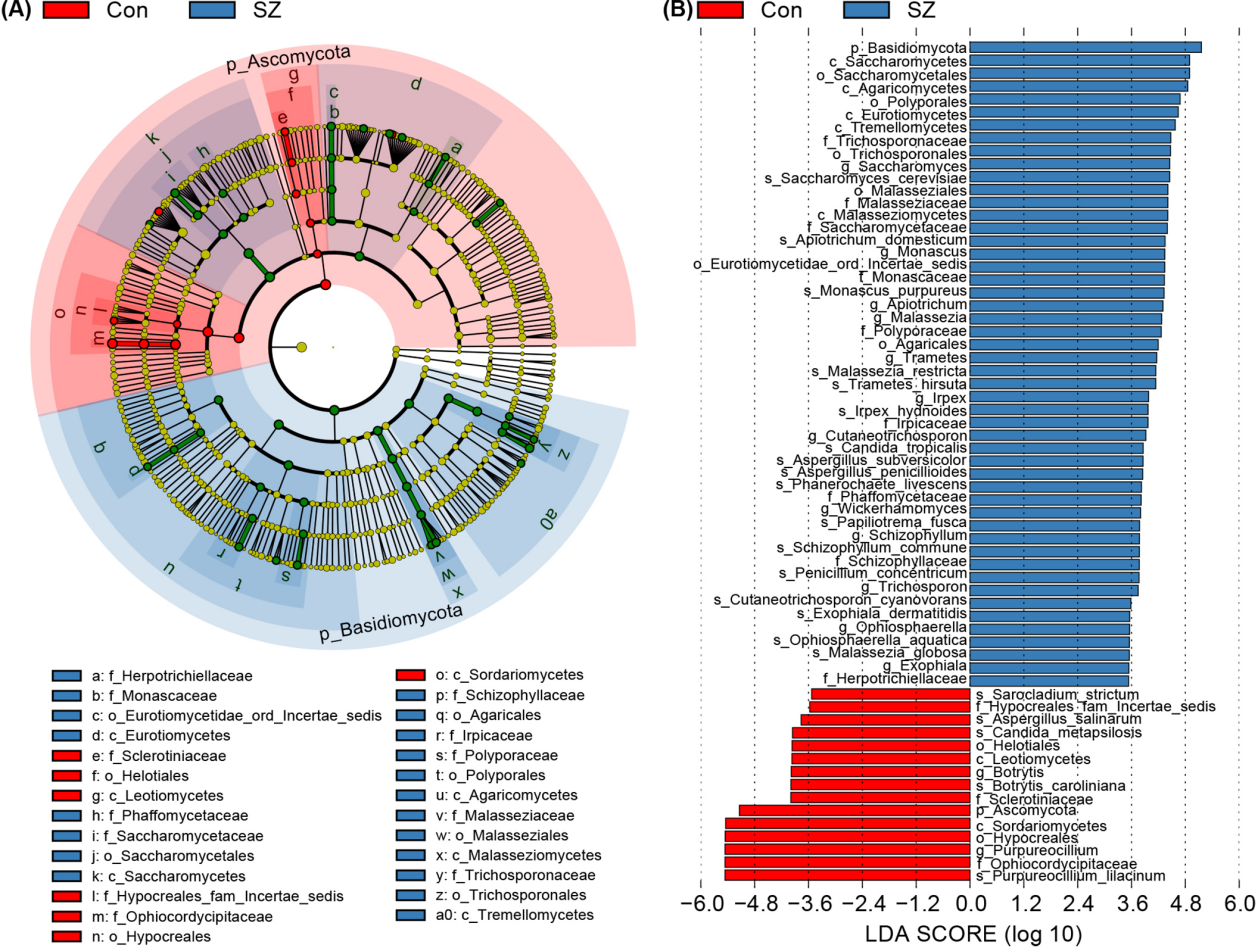
Differential fecal mycobiota between the SZ patients and healthy controls. **(A)** LEfSe cladograms illustrating fungal taxa significantly associated with SZ patients or healthy controls. The size of each circle represents the relative abundance of the fungal taxon, with circles indicating taxonomic levels from inner to outer: phylum, class, order, family, genus, and species. Statistical significance was determined using the Wilcoxon rank-sum test (p < 0.05). **(B)** Histogram displaying the distribution of Linear Discriminant Analysis (LDA) scores (> 3.5) for fungal taxa with the greatest differences in abundance between SZ patients and healthy controls (p < 0.05).

To deeply characterize the fecal mycobiota alterations in SZ, we employed the linear discriminant analysis (LDA) effect size (LEfSe) method to identify SZ-associated fecal key functional fungi. **Figure 3A** displayed cladograms that highlight differentiating fungal biomarkers from the phylum to species levels, illustrating the representation of fungal taxa between SZ patients and HCs. **Figure 3B** showed potential SZ-associated fungal biomarkers at various taxonomic levels (LDA score > 3.5, p < 0.05), with the most distinctive biomarkers at the species level including *Purpureocillium lilacinum*, *Saccharomyces cerevisiae*, *Apiotrichum domesticum*, *Malassezia restricta*, *Monascus purpureus*, *Trametes hirsute*, and *Candida tropicalis*.

**Figure 4:**
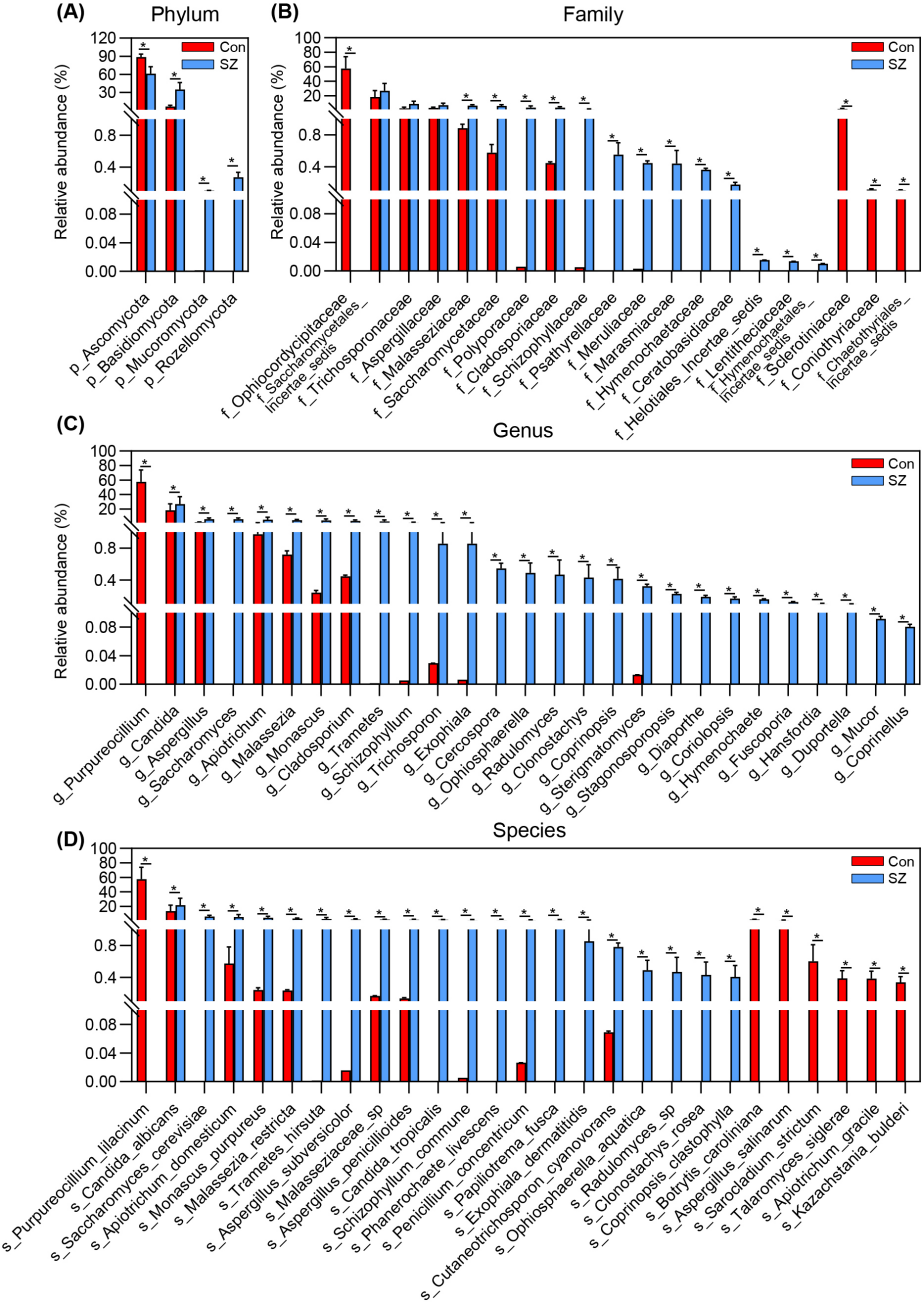
Differential fecal fungal taxa between SZ patients and healthy controls. **(A)** Key differential functional phyla; **(B)** Key differential functional families; **(C)** Key differential functional genera; **(D)** Key differential functional species. Data are presented as mean ± standard deviation. Mann–Whitney *U*-tests were used to assess differences between SZ patients and healthy controls. * p < 0.05 compared to the control group.

Then, we compared the fecal mycobiota between the two groups at specific taxonomic levels using MetaStats 2.0. At the phylum level, SZ patients exhibited an increased abundance of Basidiomycota and a decrease in Ascomycota (**Figure 4A**). The Basidiomycota/Ascomycota ratio was significantly higher in SZ patients, which may serve as a potential indicator of mycobiota dysbiosis. At the family level, 13 families including Malasseziaceae, Saccharomycetaceae, Polyporaceae, Cladosporiaceae, and Schizophyllaceae were elevated in SZ patients, and other four families such as Ophiocordycipitaceae, Sclerotiniaceae, Coniothyriaceae, and Chaetothyriales_Incertae_sedis were significantly reduced (**Figure 4B**). At the genus level, 26 genera including *Candida*, *Aspergillus*, *Saccharomyces*, *Apiotrichum* and *Malassezia* were more abundant in SZ patients, while *Purpureocillium* was almost diminished (**Figure 4C**). At the species level, *C. albicans*, *S. cerevisiae*, *A. domesticum*, *M. purpureus*, and *M. restricta* were more abundant in SZ patients, whereas species such as *P. lilacinum*, *Botrytis caroliniana*, *Aspergillus salinarum*, and *Sarocladium strictum* were significantly reduced (**Figure 4D**).

**Figure 5:**
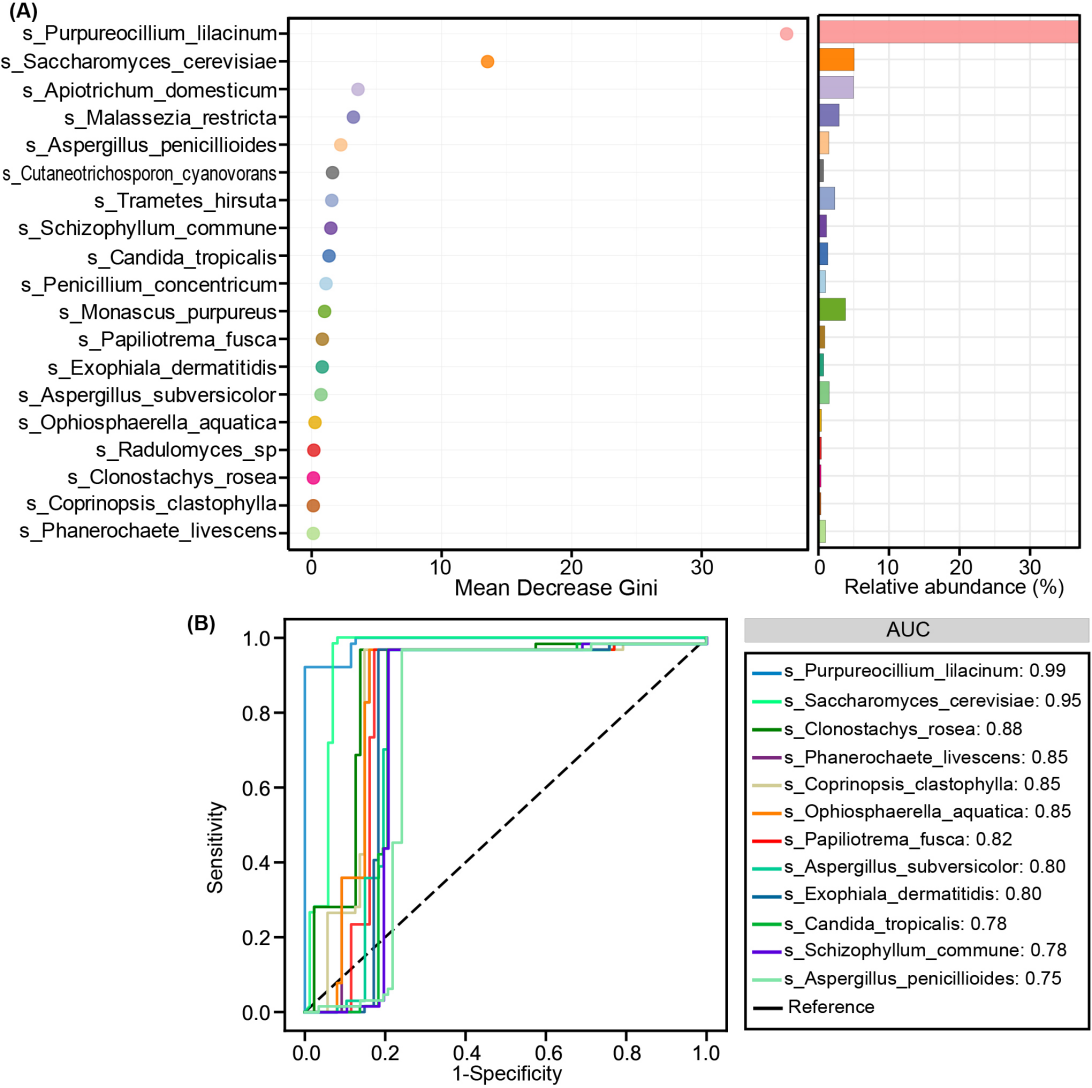
Diagnostic potential of differential fungal species in SZ patients. **(A)** Random Forest analysis with Mean Decrease Gini indicating the importance of different fungal species. **(B)** Receiver-operating characteristic (ROC) curves for individual fungal species to distinguish SZ patients from healthy controls. AUC represents the area under the ROC curve.

We evaluated the potential of key functional fungal species in distinguishing SZ patients from HCs using Random Forest and ROC analysis. Random Forest is a machine learning classification model that can efficiently and accurately classify microbial community samples. Mean Decrease Gini is used to compare the importance of variables by calculating their effect on the heterogeneity of the classification model. A higher value indicates that the key species is more important (**Figure 5A**). ROC curves were then generated to assess the diagnostic performance of these fungal species at the species level, with area under the curve (AUC) values reflecting their accuracy. Among the differential species, *P. lilacinum* (AUC = 0.99), *S. cerevisiae* (AUC = 0.95), *Clonostachys rosea* (AUC = 0.88), *Phanerochaete livescens* (AUC = 0.85), *Coprinopsis clastophylla* (AUC = 0.85), and *Ophiosphaerella aquatica* (AUC = 0.85) demonstrated significant discriminatory power (**Figure 5B**). These findings suggest that the identified fecal fungal species could serve as potential non-invasive biomarkers for diagnosing SZ in comparison to HCs.

### Altered functional traits of fecal mycobiota in SZ patients

**Figure 6:**
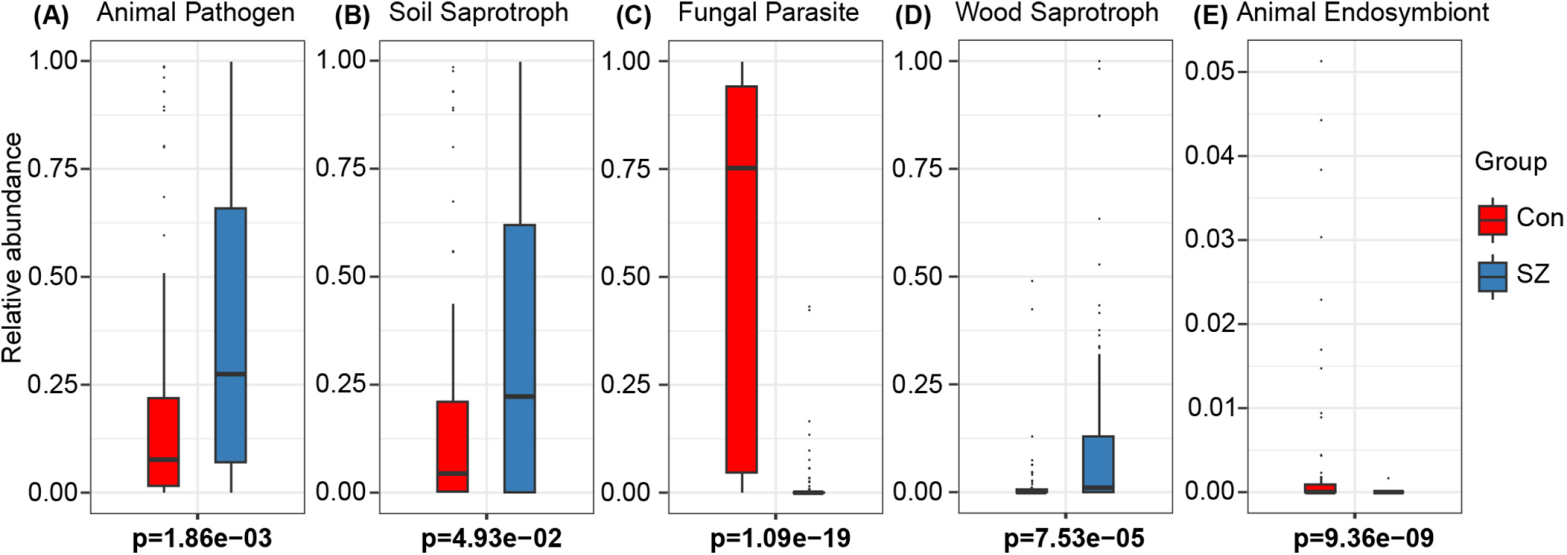
Functional prediction of nutritional modes of fungi in the fecal mycobiota of SZ patents. **(A)** Animal Pathogen; **(B)** Soil Saprotroph; **(C)** Fungal Parasite; **(D)** Wood Saprotroph; **(E)** Animal Endosymbiont.

Using PiCRUSt2, we compared enzyme classification (EC) numbers and MetaCyc pathway predictions between the two groups but found no significant differences. To further assess fungal functional roles, we employed FUNGuild, a comprehensive database that categorizes fungal taxa into distinct functional guilds based on their trophic strategies and ecological roles. Fungal taxa were classified into five functional guilds: animal pathogen, soil saprotroph, fungal parasite, wood saprotroph, and animal endosymbiont. Notably, the SZ-associated mycobiome exhibited an increased abundance of animal pathogens, fungal parasites, and wood saprotrophs, while soil saprotrophs and animal endosymbionts were reduced (**Figure 6**). These shifts in functional guild composition suggest potential gut mycobiome-host interactions that may influence SZ pathology.

**Figure 7:**
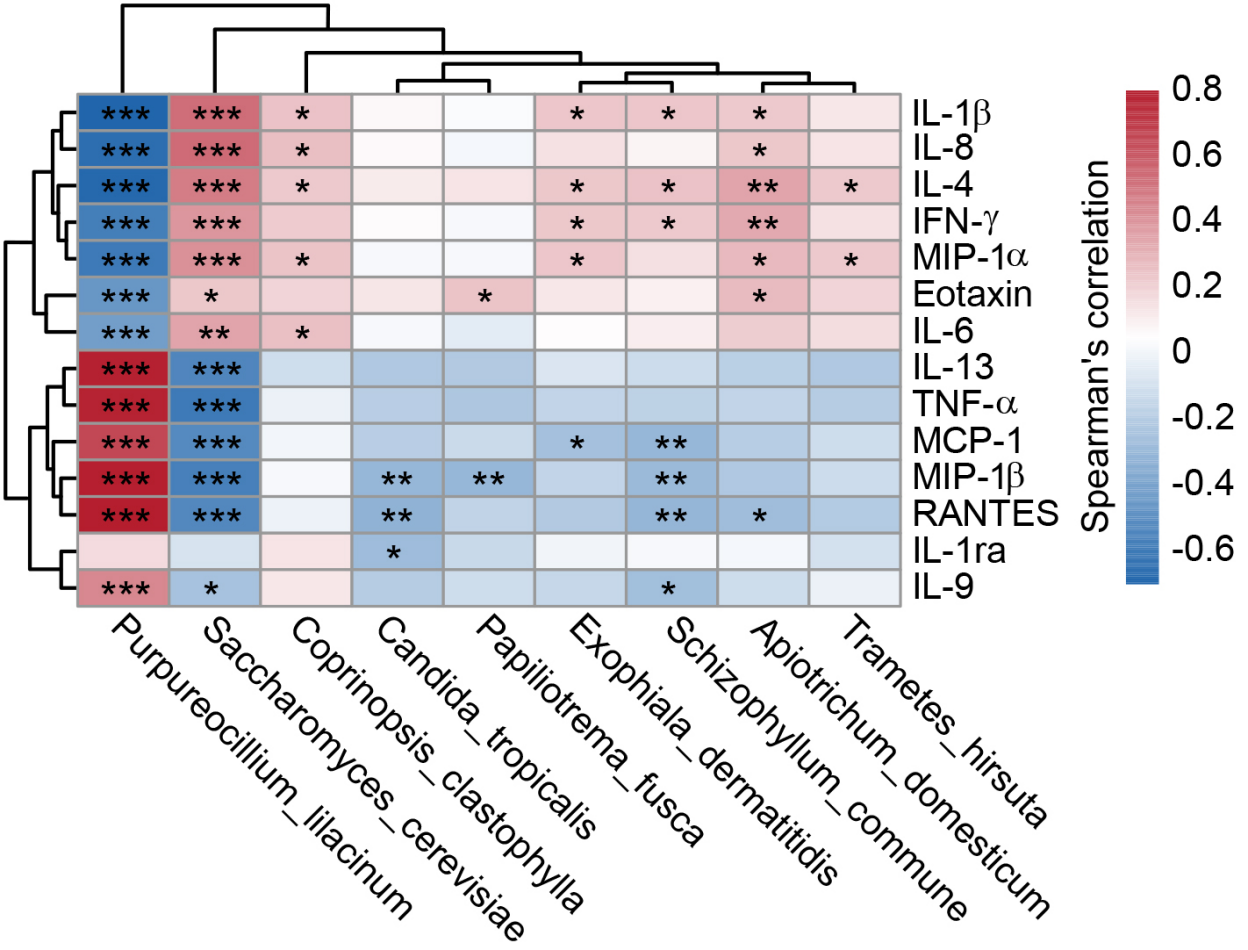
Correlations between fecal differential fungal species and systemic immune dysfunction in SZ patients. The heatmap illustrates Spearman’s rank correlations (r) and associated p-values between key functional differential species of gut mycobiota and circulating inflammatory cytokines, chemokines, and growth factors in SZ patients. Significant correlations (*p < 0.05; **p < 0.01; ***p < 0.001) are indicated.

Additionally, multiplex cytokine analysis revealed significant elevations in Eotaxin, IL-1β, IL-4, IL-6, IL-8, MIP-1α, and TNF-α in SZ patients, whereas IFN-γ, IL-9, IL-1ra, IL-13, MCP-1, MIP-1β, and RANTES were reduced (p < 0.05), indicating a complex immune dysfunction associated with SZ. Spearman’s correlation analysis identified two distinct clusters linking key fungal species with altered cytokines (**Figure 7**). Cluster I demonstrated that SZ-enriched species, such as *S. cerevisiae*, were positively correlated with elevated cytokine levels, whereas SZ-reduced species, like *P. lilacinum*, exhibited negative correlations with these cytokines. In contrast, cluster II highlighted that *P. lilacinum* was positively correlated with reduced cytokines, while *S. cerevisiae* showed negative correlations these cytokines. These findings suggest that SZ-associated fungal taxa may contribute to immune dysfunction, potentially influencing SZ pathogenesis through host-mycobiota-immune interactions.

## Discussion

Fungi represent the second-largest microbial group in the human microbiome, yet they have traditionally received less attention than bacteria in microbiome research (25). Historically, studies of the gut microbiome have primarily focused on bacterial communities, while the role of fungi has remained underexplored. This disparity in focus arises partly from the lower abundance of fungi in the gut compared to bacteria, as well as the technical challenges associated with culturing fungi and the complexities involved in their molecular and phylogenetic characterization (26). These factors have contributed to the relative underrepresentation of fungi in early gut microbiome studies (27). Nevertheless, recent advancements in molecular techniques, particularly high-throughput next-generation sequencing, have facilitated a more comprehensive investigation of the gut mycobiota (28). Specifically, ITS sequencing has allowed for more accurate assessments of fungal diversity and function within the gut, offering new insights into the role of the mycobiota in health and disease (29). Emerging research utilizing ITS sequencing has underscored the crucial role that the gut mycobiota plays in maintaining both physical and mental health (5, 7, 14, 30, 31). Fungal species within the gut microbiome have been shown to influence a variety of physiological processes, including immune regulation, metabolic pathways, and neurological function. Dysbiosis, or an imbalance in the gut mycobiota, has been linked to several health conditions, ranging from gastrointestinal diseases and metabolic disorders to neuropsychiatric conditions such as Alzheimer’s disease (AD), autism spectrum disorders, and Rett syndrome (5, 18, 30, 32–34). Alterations in the gut mycobiota can contribute to the pathogenesis of these diseases through several mechanisms, including disruption of immune responses, impairment of gut barrier integrity, and modulation of neuroactive substances. In our prior research, we identified fungal species such as *C. tropicalis* and *S. commune* as being enriched in AD patients, suggesting that these species may play a role in modulating host immunity and influencing neurological function (18). While previous studies have reported gut mycobiota dysbiosis in Chinese adult SZ cohorts (35, 36), the role of the gut mycobiota in elderly SZ patients remains largely unexplored. Given the age-related changes in the gut microbiome and immune system, it is critical to extend this line of research to understand how the gut mycobiota may influence the onset and progression of SZ in elderly.

Our study represents a comprehensive investigation into the roles of the gut mycobiota in SZ, particularly in elderly patients, revealing significant alterations in fungal composition, diversity, and functional potential that correlate with systemic immune dysfunction. Contrary to typical bacterial dysbiosis patterns in SZ, which often show reduced diversity (9), we observed elevated α-diversity (Shannon, Simpson) and richness (Chao1, ACE) in the gut mycobiota of SZ patients. This divergence suggests that fungal and bacterial communities may respond differently to disease-associated perturbations. The underlying mechanisms for this divergent response remain unclear but could be related to the specific ecological niches occupied by fungi and bacteria in the gut, as well as their differing interactions with the host immune system. Dollive et al. has found that antibiotic treatment increases the fungal diversity in mice, which was reversed after treatment interruption (37). This highlights the dynamic nature of the gut mycobiota and suggests that fungi and bacteria are not isolated entities but rather parts of a larger, interconnected ecosystem. Interestingly, decreased gut fungal diversity has been reported in adult SZ patients (35, 36), which aligns with the bacterial dysbiosis seen in SZ. Shuai et al. demonstrated that age plays a key role in shaping gut fungal composition, with age being the most significant factor in interindividual variation in the gut mycobiota, suggesting that some fungal taxa change over time (38). The dynamic age-related gut mycobiota changes highlight the need to explore key fungi more carefully. The differences observed between adult and elderly SZ patients in our study may reflect the influence of aging, suggesting that age-related factors may modulate the role of the gut mycobiota in SZ pathophysiology.

In our elderly cohort, we observed a notable dysbiosis in the fecal mycobiota of SZ patients, characterized by an elevated Basidiomycota/Ascomycota ratio. This imbalance in fungal composition suggests potential alterations in the gut mycobiota that could be linked to SZ pathology. Similar shifts in the Basidiomycota/Ascomycota ratio have been reported in other neurological and gastrointestinal disorders, such as multiple sclerosis, Parkinson’s disease (PD), irritable bowel syndrome (IBS), and inflammatory bowel disease (IBD) (39–43). The overrepresentation of *Candida*, *Saccharomyces* and *Aspergillus*, along with a depletion of *Purpureocillium*, may disrupt gut homeostasis in SZ patients, potentially exacerbating gut permeability and promoting systemic inflammation. Immune dysregulation has long been proposed as a key environmental factor in the pathogenesis of SZ. Recent advances in the immunopsychiatric field have provided compelling evidence for the involvement of both innate and adaptive immune components in SZ (44). Our immunological analysis reveals complex disturbances in elderly SZ patients, suggesting that chronic, low-grade inflammation may impair cognitive function in this population (45, 46). Notably, the reduced abundance of *P. lilacinum* in SZ patients, correlated with pro-inflammatory cytokines like TNF-α, MCP-1 and MIP-1β, indicates its potential role in immune modulation. Interestingly, our previous oral mycobiota study in these patients showed opposite associations, where *P. lilacinum* positively correlated with inflammatory markers such as TNF-α, MIP-1α and Eotaxin (15). Yuan et al. further suggested that elevated *Purpureocillium* abundance in adults with SZ was linked to worse cognitive function, potentially mediated by metabolites like leucinostatin Y, which could disrupt amino acid metabolism and neuroinflammation (36, 47). While *P. lilacinum* is often considered opportunistic pathogenic, its production of purlisin, a defensin with immunosuppressive and antibacterial properties, complicates its role in SZ (48). Additionally, secondary metabolites like hydroxybenzoic acids demonstrate anti-inflammatory and immunomodulatory properties (49). These contrasting findings likely reflect tissue-specific and age-dependent effects, with *P. lilacinum* potentially serving protective roles in the elderly gut while contributing to mucosal defense in the oral cavity. Enterotype analysis further revealed a shift from *Purpureocillium*-dominant (E1) to *Candida*-dominant (E2) communities in SZ, which could influence disease progression and symptomatology (50). Several *Candida* species are known to be pathogenic and can contribute to systemic inflammation (51, 52). For instance, *C. albicans* has been associated with gut dysbiosis and various inflammatory conditions (51). It activates the NF-κB and MAPK signaling pathways via upregulating expression of a small secreted cysteine-rich protein Sel1, inducing the expression of proinflammatory cytokines and chemokines (53). Elevated *C. albicans* in SZ patients has been linked to worsened psychiatric symptoms (54), with probiotic treatment potentially alleviating symptoms by reducing *C. albicans* antibody levels (55). This association underscores the potential therapeutic value of modulating the gut mycobiota for improving clinical outcomes in SZ. Our findings diverge from previous studies in adult SZ patients (35, 36), representing a novel observation specifically in elderly SZ patients. These age-dependent differences may reflect variations in immune function, gut motility, and dietary habits, suggesting that aging may make elderly SZ patients more vulnerable to specific fungal dysbiosis or exacerbate pre-existing vulnerabilities.

Using LEfSe and MetaStats, key functional fungi were identified within the gut mycobiota associated with SZ. Specifically, *S. cerevisiae*, *A. domesticum*, *M. purpureus*, and *M. restricta* were enriched in SZ patients, while *B. caroliniana*, *A. salinarum*, and *S. strictum* were significantly reduced. Despite these findings, the precise roles of most of these fungi in SZ pathophysiology remain largely undefined and warrant further exploration. *S. cerevisiae* is generally considered a beneficial microorganism, commonly used as a probiotic and food additive. However, our study found significant enrichment of *S. cerevisiae* in SZ patients, aligning with previous research showing that both *S. cerevisiae* and *C. albicans* are highly prevalent in the mucosa and feces of patients with IBD, especially in those with Crohn’s disease (CD) (56, 57). The colonization of *S. cerevisiae* can trigger immune responses, leading to the production of anti-*S. cerevisiae* antibodies (ASCA), which are used as biomarkers for distinguishing between CD and ulcerative colitis (UC), as well as for monitoring IBD progression (58). In our study, *S. cerevisiae* was correlated with elevated pro-inflammatory cytokines, including Eotaxin, IL-1β, IL-4, IL-6, IL-8, MIP-1α, and TNF-α, all of which are associated with immune activation and inflammation—key features of immune dysregulation in SZ. Moreover, Galimberti et al. indicated that elevated levels of anti-*S. cerevisiae* antibodies, which are linked to gut permeability, are associated with an increased risk of SZ (59). These antibodies are considered a marker of intestinal inflammation, with particularly high levels observed in individuals experiencing recent-onset SZ (60–62). These findings suggest that *S. cerevisiae* may play a context-dependent role in SZ pathogenesis, potentially contributing to immune dysregulation in genetically or environmentally susceptible individuals. Our findings underscore the complex involvement of gut microbiota in SZ and propose that *S. cerevisiae* may act as a mediator of immune dysregulation, challenging the traditional view of it as a purely beneficial microorganism. Previous studies have also demonstrated that the increased abundance of *C. albicans* and *S. cerevisiae* is positively correlated with the severity of SZ symptoms (55). However, in contrast to previous reports, *C. albicans* was not correlated with inflammatory mediators in SZ patients. Instead, *C. tropicalis* was negatively correlated with chemokines such as MIP-1β, RNATES, and anti-inflammatory cytokines like IL-1ra. Various strains of *C. tropicalis*, isolated from environmental, industrial, and clinical settings, may exhibit distinct behaviors based on their source. Doan et al. recently observed a novel role for commensal *C. tropicalis* in resolving intestinal inflammation through vitamin B3 metabolism modulation using its nicotinamidase in a strains-dependent manner (63). Notably, the *C. tropicalis* strain MYA-3404, but not clinical isolates from Crohn’s disease patients, is a potent inducer of IL-17A from Th17 and γδ T cells, as well as IL-22 from ILC3s, thereby enhancing intestinal barrier function by promoting epithelial cell proliferation and inducing goblet cell differentiation (63). Furthermore, Roberts et al. observed that *C. tropicalis* may produce molecules that inhibit *C. albicans* adhesion to the intestinal surface, reducing biofilm formation (64). Additionally, *M. restricta*, a common saprophytic gut pathogen, was found to promote the secretion of Th2-type cytokines upon its presence (65), which may potentially influence the prognosis and severity of SZ (66). Phuna et al. demonstrated that *M. restricta* can induce neuroinflammation by activating helper Th1 and Th17 immune responses in AD mice (67). ITS sequencing has identified *Malassezia* DNA in the brains of patients with AD, multiple sclerosis (MS), and amyotrophic lateral sclerosis (ALS) (68–70), further suggesting its potential involvement in neurological conditions. Moreover, the presence of *Malassezia* has been suggested in relation to PD (71), and confirming this link could allow for the examination of anti-*Malassezia* antibody titers as potential clinical biomarkers (72). However, the exact role of *Malassezia* in these disorders remains unclear, but its ability to infect human cells, enter the bloodstream, cross the blood-brain barrier, and coexist with lipid-rich neuronal cells may contribute to its pathogenic mechanisms. An increased abundance of *Aspergillus* has been observed in the fecal and oral mycobiota of elderly SZ patient, while a decrease is noted in adult patients (73). Yuan et al. observed a positive correlation between *Aspergillus* and cognitive function, with its protective effect against SZ attributed to chitin-mediated anti-inflammatory properties through the induction of IL-1ra (47). They also observed that a lower abundance of *Aspergillus* and a higher abundance of *Megasphaera* may contribute to an upregulated inflammatory status in SZ patients (36). Contrary to these findings in adult patients, the increased *Aspergillus* abundance in elderly patients was not correlated with inflammatory indicators, which might be due to age-related differences in immune response or altered gut microbiota composition in the elderly population. These results indicate that the composition of the gut mycobiota, specifically the presence of species like *S. cerevisiae* and *P. lilacinum*, may influence the immune system in SZ patients, potentially contributing to the pathology of the disorder through complex host-mycobiota-immune interactions.

Although the precise mechanistic roles of these key functional fungi in SZ pathogenesis remain incompletely understood (74), our ROC analysis identified several species with exceptional discriminatory power (AUC > 0.85), suggesting their potential as non-invasive diagnostic biomarkers or therapeutic targets (75, 76). Most notably, *P. lilacinum* (AUC = 0.99) demonstrated near-perfect classification accuracy, with its dramatic depletion in SZ patients correlating strongly with elevated pro-inflammatory cytokines and reduced anti-inflammatory markers. This suggests *P. lilacinum* may serve a protective, immunomodulatory role in gut-brain axis homeostasis, possibly through its known antimicrobial properties that suppress pathogenic overgrowth. Conversely, the enrichment of *S. cerevisiae* (AUC = 0.95) and its positive association with pro-inflammatory cytokines (IL-1β, IL-8) aligns with emerging evidence of fungal-induced gut barrier disruption and autoantibody production in SZ. The divergent correlations of *C*. *tropicalis* (reduced IL-1ra/MIP-1β despite overall enrichment) further underscore the complex, species-specific interactions between fungi and host immunity. Our previous study on oral mycobiota also demonstrated that *C. albicans* and *M. restricta* possess discriminatory potential in distinguishing SZ patients from HCs (15). These ROC-driven insights not only position specific fungal taxa as robust classifiers of SZ but also suggest their functional involvement in disease-related immune dysregulation - whether as primary drivers or secondary amplifiers of pathology. Importantly, the high predictive accuracy of these mycobiota signatures (particularly *P. lilacinum*) supports their potential utility in clinical stratification, while their mechanistic links to inflammation offer testable hypotheses for mycobiota-targeted interventions, such as probiotic restoration of commensal fungi or antifungal modulation of pathobionts.

However, our study is not without limitations. First, although it highlights the interactions between the mycobiota and the immune system in SZ, causality remains unclear. The cross-sectional design precludes the establishment of definitive causal relationships. Longitudinal studies are necessary to elucidate the temporal dynamics of the gut mycobiota and its role in the onset and progression of SZ. Second, mechanistic experiments using animal models could provide insights into how specific fungal species modulate neuroinflammation. Future research should prioritize longitudinal investigations to determine whether these fungal alterations precede disease onset and to explore their causal roles through gnotobiotic models. Third, the interplay between the gut mycobiota and other microbial communities, including bacteria and viruses, requires further exploration. A more comprehensive understanding of these microbial populations will be crucial in elucidating their collective impact on mental health.

In conclusion, our study provides a comprehensive characterization of the significant alterations in fungal diversity, composition, and functional potential that are associated with systemic immune dysfunction in elderly individuals with SZ. These findings offer valuable insights into the potential role of the gut mycobiota in SZ pathophysiology. Notably, we observed a shift in the SZ-associated gut mycobiota from a *Purpureocillium*-dominant enterotype to a *Candida*-dominant profile. This suggests that specific gut fungi, particularly *Purpureocillium* and *Saccharomyces*, may play a critical role in modulating host immune responses, thereby contributing to the onset and progression of SZ. Our results expand the gut-brain axis paradigm, highlighting these key fungal species as potential diagnostic biomarkers and therapeutic targets. Further research into the interactions between the mycobiota and the immune system, as well as the development of strategies to modulate the mycobiota, could pave the way for novel therapeutic approaches for SZ and other neuropsychiatric disorders.

## Data Availability

The datasets presented in this study can be found in online repositories. The names of the repository/repositories and accession number(s) can be found below: https://www.ncbi.nlm.nih.gov/, PRJNA1243042.
